# NfL as a biomarker for neurodegeneration and survival in Parkinson disease

**DOI:** 10.1212/WNL.0000000000010084

**Published:** 2020-08-18

**Authors:** David Bäckström, Jan Linder, Susanna Jakobson Mo, Katrine Riklund, Henrik Zetterberg, Kaj Blennow, Lars Forsgren, Niklas Lenfeldt

**Affiliations:** From the Department of Clinical Science (D.B., J.L., L.F., N.L.), Neurosciences, and Department of Radiation Sciences (S.J.M., K.R.), Diagnostic Radiology and Umeå Center for Functional Brain Imaging, Umeå University; Department of Psychiatry and Neurochemistry (H.Z., K.B.), Institute of Neuroscience and Physiology, the Sahlgrenska Academy at the University of Gothenburg; Clinical Neurochemistry Laboratory (H.Z., K.B.), Sahlgrenska University Hospital, Mölndal, Sweden; Department of Neurodegenerative Disease (H.Z.), UCL Queen Square Institute of Neurology; and UK Dementia Research Institute at UCL (H.Z.), London, UK.

## Abstract

**Objective:**

To determine whether neurofilament light chain protein in CSF (cNfL), a sensitive biomarker of neuroaxonal damage, reflects disease severity or can predict survival in Parkinson disease (PD).

**Methods:**

We investigated whether disease severity, phenotype, or survival in patients with new-onset PD correlates with cNfL concentrations around the time of diagnosis in the population-based New Parkinsonism in Umeå (NYPUM) study cohort (n = 99). A second, larger new-onset PD cohort (n = 194) was used for independent validation. Association of brain pathology with the cNfL concentration was examined with striatal dopamine transporter imaging and repeated diffusion tensor imaging at baseline and 1 and 3 years.

**Results:**

Higher cNfL in the early phase of PD was associated with greater severity of all cardinal motor symptoms except tremor in both cohorts and with shorter survival and impaired olfaction. cNfL concentrations above the median of 903 ng/L conferred an overall 5.8 times increased hazard of death during follow-up. After adjustment for age and sex, higher cNfL correlated with striatal dopamine transporter uptake deficits and lower fractional anisotropy in diffusion tensor imaging of several axonal tracts.

**Conclusions:**

cNfL shows usefulness as a biomarker of disease severity and to predict survival in PD. The present results indicate that the cNfL concentration reflects the intensity of the neurodegenerative process, which could be important in future clinical trials.

**Classification of evidence:**

This study provides Class II evidence that in patients with PD, cNfL concentrations are associated with more severe disease and shorter survival.

The prognosis, life expectancy, and clinical presentation in Parkinson disease (PD) are highly variable.^1–4^ For example, patients with PD who present with resting tremor have milder disease and longer survival compared to patients who present with gait disturbance.^4–6^ Insufficient understanding of the mechanisms driving neurodegeneration and the lack of biomarkers to dynamically measure their rate hamper the development of disease-modifying therapies for PD.

A promising biomarker in neurodegenerative diseases is the neurofilament light chain protein (NfL), which provides a sensitive measurement of neuroaxonal damage, regardless of cause.^7^ NfL in CSF (cNfL) was higher in patients with PD compared with healthy controls in some but not all studies.^8,9^ While many patients with PD have normal levels,^10^ we previously showed that high cNfL in early PD predicts later progression to PD dementia.^8^ In animal models of PD, experimental induction of α-synuclein deposition in neurons is associated with marked increases of cNfL.^11^ Higher cNfL levels might be explained by a more aggressive and widespread neurodegeneration in a subset of patients with PD.

Among idiopathic parkinsonian disorders, increased cNfL is particularly evident in multiple system atrophy, progressive supranuclear palsy, and corticobasal syndrome,^9,12,13^ i.e., diseases with more extensive neurodegeneration than typical PD. Technological advancement has now allowed sensitive quantification of NfL in blood, which may allow accessible monitoring.^7^ However, NfL is not well studied as a biomarker of neurodegeneration in PD. The primary aim of this study was to investigate whether the severity of PD is related to cNfL levels and whether cNfL levels predict survival. To further investigate possible correlation with neurodegeneration, striatal dopamine transporter (DAT) imaging and specific symptoms such as hyposmia were also studied in relation to cNfL as secondary endpoints. Finally, because magnetic resonance diffusion tensor imaging (DTI) is a sensitive marker for axonal tract damage in early-stage PD^14^ and may expose regional patterns of disease pathology in relation to cNfL increases, we repeated DTI in the population-based PD cohort.

## Methods

### Study populations

All patients in the original cohort participated in a population-based incidence study of unselected cases of new-onset idiopathic parkinsonism from a geographic catchment area of ≈142,000 inhabitants in northern Sweden (the New Parkinsonism in Umeå [NYPUM] study). The inclusion period was between January 1, 2004, and April 30, 2009. A population screening procedure was performed to avoid selection bias and to make PD case identification as complete as possible.^15^ Patients with atypical parkinsonism (e.g., multiple system atrophy or progressive supranuclear palsy, n = 31), secondary parkinsonism (e.g., drug-induced parkinsonism or stroke), or dementia at baseline, defined by an Mini Mental State Examination score <24 and clinical symptoms, were excluded. These patients are described in previous publications.^4,8^ Patients with incident PD (n = 143) were included and followed up prospectively with standardized clinical examinations, including the modified Hoehn and Yahr (HY) and Unified Parkinson's Disease Rating Scale (UPDRS), at least yearly. Medication was documented by levodopa equivalent daily dose. MRI and presynaptic imaging of DAT status with ^123^I-FP-CIT SPECT were done repeatedly. Of the 143 enrolled patients with PD, 99 agreed to CSF collection by lumbar puncture at study entry (baseline). The patients who declined collection of CSF (n = 44) were older than the patients who participated (74.3 vs 69.8 years) but had comparable scores for mood, motor, and cognitive dysfunction.

For validation, a second cohort consisting of all patients with new-onset, idiopathic parkinsonism referred from primary care to the neurologic department at Umeå University Hospital (which is the only neurologic department in the area) from May 2009 to September 2018 (the validation cohort) was investigated. All patients with PD were offered a lumbar puncture for CSF analysis around the time of diagnosis. One hundred ninety-four patients with new-onset PD agreed to perform CSF collection and were included. In agreement with the exclusion criteria in the original cohort (the NYPUM cohort), patients with secondary parkinsonism, atypical parkinsonism, or dementia at baseline were excluded. All patients were investigated by neurologic examination and motor assessments at baseline. In both cohorts, a diagnosis of PD required agreement among the examiners (neurologists specialized in movement disorders) that the clinical criteria for the diagnosis were fulfilled according to the UK PD Society Brain Bank criteria.^16^ The diagnosis of PD was neuropathologically verified at autopsy in 6 cases in the population-based cohort.

For comparison, 30 healthy control participants that were age- and sex-matched to the first 50 patients included in the NYPUM cohort were recruited. Controls were recruited by advertisements in the local newspaper, and in a few cases among friends and family of the PD participants. Requirements for controls were that they had no neurological disorders, had a normal neurological exam, and normal 123I-FP-CIT SPECT brain imaging.

### Standard protocol approvals, registrations, and patient consents

All participants provided informed consent. The study was approved by the Regional Medical Ethics Board in Umeå, Sweden (Um dnr 03-387, dnr 2014-163-31M) and was performed in accord with the Declaration of Helsinki.

### Clinical evaluation

In both cohorts, motor function was assessed in the early phase of PD, before dopaminergic medication was started at baseline, with the HY scale and the UPDRS. In the NYPUM cohort, the UPDRS scores were divided into subscores for tremor (sum of items 20 and 21) and postural imbalance and gait difficulty (PIGD; the sum of items 13–15, 29, and 30).^17^ Bradykinesia (measured by the sum of UPDRS items 23–26 and 31) and axial symptoms (measured by the sum of UPDRS items 18, 27–29, and the neck rigidity of item 22) were also investigated. Data on UPDRS subscales were lacking in the validation cohort. Instead, the most predominating symptom that first led the patient to contact a health care service was recorded and classified as (1) resting tremor, (2) bradykinesia (including, e.g., micrographia and clumsiness) (3) balance impairment or gait difficulty (e.g., shuffling gait, freezing of gait, or postural imbalance), or (4) other symptom. In the NYPUM cohort, olfactory function was tested at baseline and after 1 year by the 12-item Brief Smell Identification Test.^18^ Mobility was measured by the Timed Up and Go test, which is the time it takes to rise up from a chair, walk 3 m, and sit down again.^19^

### Survival

After inclusion, all surviving patients in the population-based cohort (NYPUM) were followed up yearly for ≈8.5 to 13.5 years, until August 31, 2017. The surviving patients in the validation cohort were followed up until October 31, 2018. The survival data were complete for both cohorts. All-cause mortality was studied as the relevant outcome.

### Imaging with SPECT and CSF analysis

All 99 patients with PD from whom CSF was collected in the NYPUM cohort, all 194 patients in the validation cohort, and 30 age-matched healthy controls underwent presynaptic DAT imaging by ^123^I-FP-CIT (DaTSCAN; GE Healthcare BV, Eindhoven, the Netherlands) SPECT at baseline. All patients had a pathologic scan. The DAT imaging protocol in the population-based study (NYPUM) was performed within a nonprofit trial (EU No. 2009-011748-20) and constituted a substudy within the research project. Semiquantitative analysis (based on regions of interest) and visual evaluation of the DAT SPECT were done unbiased by clinical information. Normal reference values were derived from the healthy control participants,^20,21^ and reduction of DAT uptake in the putamen and caudate of the patients with PD was measured in SDs of the normal values. The imaging protocol, equipment, and semiquantitative evaluation methods were described earlier.^20^ Two different SPECT cameras were used during the course of the population-based study; 1 brain-dedicated SPECT camera (the Neurocam) was later substituted by a multipurpose hybrid SPECT/CT (both General Electric, Milwaukee, WI). Twenty-two patients performed DAT imaging in the Neurocam and 77 in the hybrid SPECT/CT.

Standard procedures for lumbar puncture for collection of CSF were used, with the patient lying in the decubitus position. In the validation cohort, the lumbar puncture was generally performed somewhat later than in the NYPUM study, within 2 to 3 months from the first (baseline/diagnostic) visit. While on dopaminergic treatment, CSF collection was repeated in the NYPUM cohort after 1 and 3 years in 53 and 35 patients, respectively. The patients who participated in repeat CSF collection had a lower HY stage than the other patients with PD (1.9 vs 2.3 at 1 year) but comparable ages and UPDRS scores. CSF levels of NfL were measured with a sandwich ELISA (NF-Light; UmanDiagnostics AB, Umeå, Sweden), as described by the manufacturer.^22,23^ The coefficient of variation was 14.0%. All analyses were performed by experienced, board-certified laboratory technicians using procedures approved by the Swedish Board for Accreditation and Conformity Assessment. The CSF analyses were performed by investigators blinded to all clinical data.

### Diffusion tensor imaging

In the NYPUM cohort, 45 of the 99 patients with PD from whom CSF was collected at baseline had a 3.0T MRI scan at baseline. Twenty and 14 patients had both the MRI scan and CSF collection at the 1 and 3-year follow-ups, respectively. Magnetic resonance DTI was performed with single-shot spin echo planar imaging sequences with the following parameters: repetition time shortest, echo time 77 milliseconds, flip angle 90°, field of view 224 × 224 mm, acquisition matrix 112 × 112, reconstruction matrix 256 × 256, b value 1,100 s/mm^2^, 70 slices (continuous), 16 gradients, and 2-mm slice thickness. One nongradient (B0) volume was also sampled. The extracted B0 image was used in the procedure of realignment and motion and eddy current correction,^24^ with linear/quadratic terms applied for the spatial model of the eddy current–induced field and linear terms for the model of the diffusion gradients. Postprocessing of the images included conversion from Digital Imaging and Communications in Medicine to Neuroimaging Informatics Technology Initiative format, eddy current correction, and brain extraction. The images were processed with the diffusion toolbox FSL (fsl.fmrib.ox.ac.uk/fsl/fslwiki/FDT). All images were visually checked after correction, and no sample was disregarded on the basis of improper correction. The DTI map of fractional anisotropy (FA) was generated by fitting diffusion tensors to the corrected data. The postprocessing followed the Tract-Based Spatial Statistics method, which is part of the FSL package.^25^ The most typical patient was used for normalization. Randomize (part of the FSL package) was finally used to reveal areas in the brain where FA correlated negatively with cNfL levels (i.e., lower FA correlated with higher NfL).

### Statistics

Baseline correlations between PD phenotype, severity scales, striatal DAT imaging uptake, and cNfL concentrations were tested by Spearman ρ or Pearson *r*, as appropriate. Differences in cNfL concentrations between the PD cohorts, between PD subtype groups, and between patients and controls were tested by 1-way analysis of covariance to allow adjustment for age, sex, and disease duration. These tests were conducted after log-transformation of cNfL values to obtain normal distributions. Associations between PD phenotype, severity measures, and cNfL in the larger pooled cohort (n = 293) were also analyzed with multiple linear regression, and differences per unit of change of cNfL (measured by β values) were estimated with and without adjustments. In these analyses, the normality of data was assessed by inspection of residuals. Possible change of cNfL over time was investigated by the Wilcoxon signed rank test. Cox proportional hazards regression was used to investigate whether the baseline cNfL level predicted mortality. Kaplan-Meier plots show effects on survival of a baseline cNfL concentration below/above the overall median in PD (903 ng/L) and in the highest and lowest quartiles. The predictive value of baseline cNfL levels was described by area under the receiver operating characteristic curve (AUROC), and levels below/above cutoffs with the highest Youden Index (sensitivity + specificity − 1) were investigated for all patients who were followed up until death or 5 or 8 years. To investigate cNfL in relation to striatal dopaminergic denervation, DAT-uptake ratios were normalized by average SDs above or below the normal mean (i.e., *z* score) to equate the numerical values derived from the 2 different scanners that were used. Negative correlation between white matter integrity measured by FA and the cNfL concentration was analyzed. All correlations were adjusted for age and sex. So as not to exclude possible brain regions of interest a priori, the 10 largest contiguous correlation clusters (all being >30 voxels) for each occasion (baseline, 1 year, and 3 years) where all voxels exceeded the significance threshold *p* < 0.05 (uncorrected) are reported.

Values of *p* < 0.05 were considered significant. However, in analyses with several tests (the comparisons shown in tables 1–3), Tukey honest significance difference and Holm-Bonferroni correction were applied to correct for multiple comparisons. All statistical analyses were performed with SPSS 23.0 (SPSS, Inc, Chicago, IL).

**Table 1:**
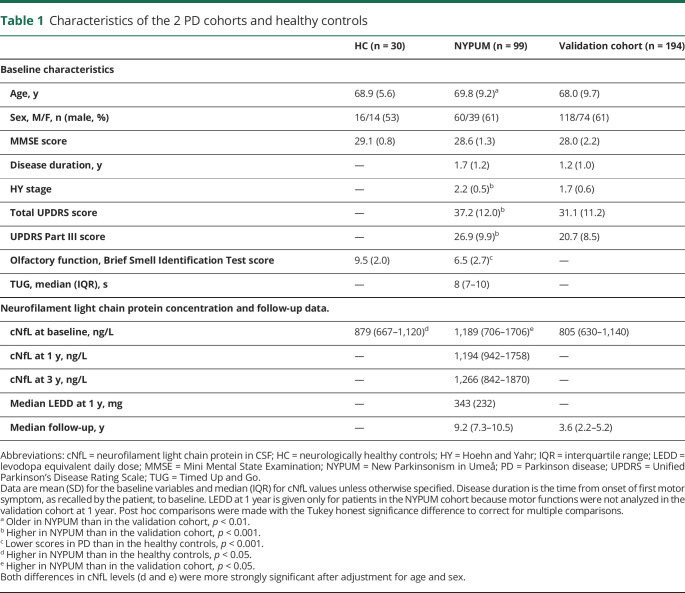
Characteristics of the 2 PD cohorts and healthy controls

**Table 2:**
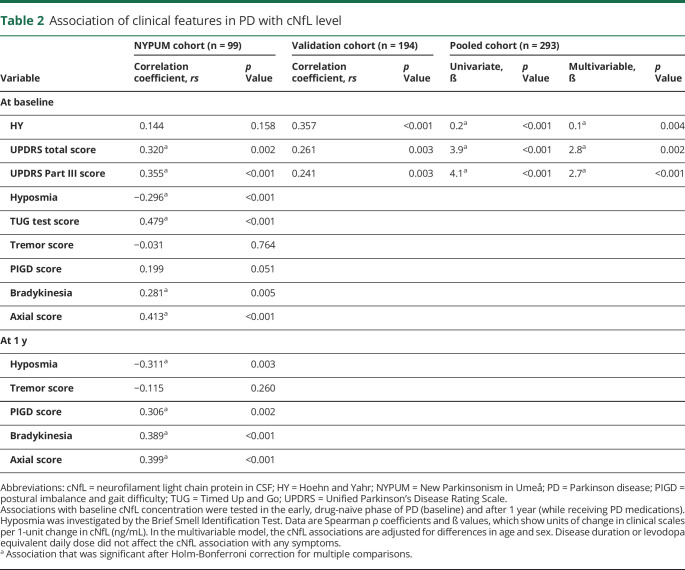
Association of clinical features in PD with cNfL level

**Table 3:**
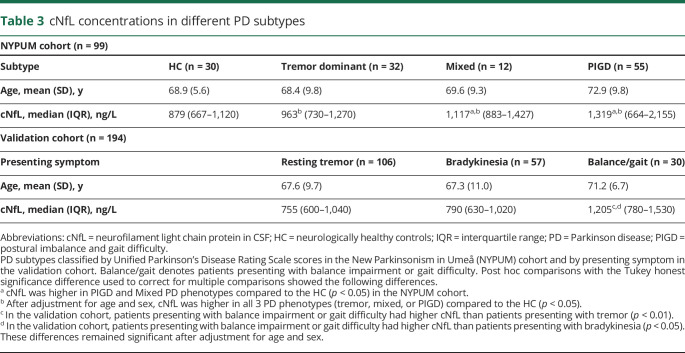
cNfL concentrations in different PD subtypes

### Data availability

Anonymized data can be obtained by request from any qualified investigator for purposes of replicating procedures and results.

## Results

### Baseline characteristics of the cohorts

**Figure 1:**
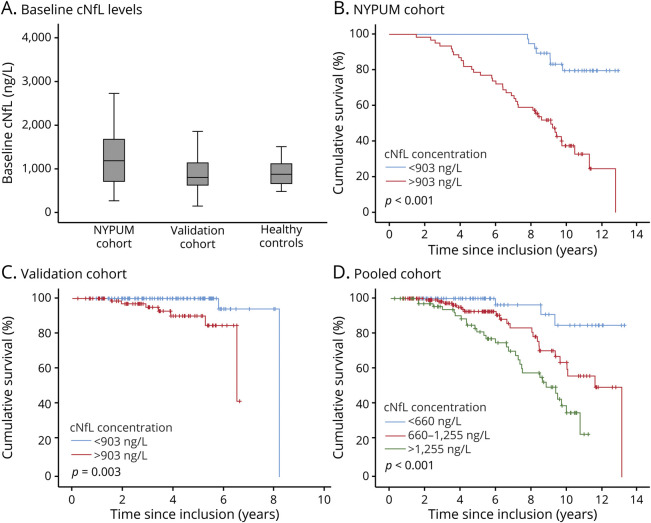
Survival in PD in relation to baseline cNfL concentration (A) Box plots of baseline neurofilament light chain protein in CSF (cNfL) levels in Parkinson disease (PD). Cumulative survival for patients with a baseline cNfL below the median concentration of 903 ng/L (blue line) compared with those with cNfL above 903 ng/L (red line) in the (B) New Parkinsonism in Umeå (NYPUM) cohort and (C) validation cohort. (D) Cumulative survival in the combined, pooled cohort for patients (n = 293) with baseline cNfL levels in the lowest (<660 ng/L; blue line) and highest (>1,255 ng/L; green line) quartiles and those with concentrations between these levels (red line).

A total of 293 patients with incident, treatment-naive PD (38.6% female and 61.4% male) and 30 neurologically healthy controls were included in this study. At baseline, there were no major demographic differences between the healthy controls and the patients with PD or between the PD patients in the population-based (NYPUM) and validation cohorts (table 1). However, the patients with PD in the population-based cohort were marginally older than the patients in the validation cohort (69.8 vs 68.0 years, *p* < 0.05) and had higher HY and UPDRS scores (*p* < 0.001) and higher cNfL concentrations (*p* < 0.05). Their cNfL concentrations were also higher compared with the healthy controls (*p* < 0.05), and this difference remained significant after adjustment for age and sex. In both PD cohorts, as well as in the controls, cNfL concentrations correlated positively with age (*r* range = 0.54–0.63). The variance of cNfL tended to be higher in PD than in the controls (figure 1A), and cNfL concentrations were similar in male and female patients (median 950 vs 798 ng/L, *p* = 0.101). In the 6 patients with an autopsy-verified diagnosis of PD (who had a mean age of 73.3 years at baseline), the baseline cNfL concentration ranged from 504 to 3,490 ng/L, and the baseline median was 1852 ng/L.

### cNfL, disease severity, and survival in PD

In both PD cohorts, the cNfL concentration correlated positively with motor symptom severity as measured by clinical scales, except for tremor (table 2), with and without adjustment for age, sex, and disease duration and (at 1 year) medication. At baseline, after additional adjustment for multiple comparisons, a higher cNfL concentration correlated positively with the total UPDRS score, the UPDRS Part III score, the bradykinesia and axial symptom subscores, the Timed Up and Go test, and the severity of hyposmia (as measured by the Brief Smell Identification Test) in the NYPUM cohort. The correlations between symptom severity and cNfL were in the range of *r* = 0.28–0.48 (*p* values between 0.005 and <0.001, table 2). Tremor showed no correlation. At the 1-year follow-up in the NYPUM cohort, a positive correlation remained between the baseline cNfL concentration and the severity of all tested symptoms (the PIGD, bradykinesia, and axial subscores and with hyposmia) except tremor. In the validation cohort, a positive cNfL correlation was confirmed for the UPDRS Part III and total UPDRS scores, and there was a significant correlation with the HY stage. In the NYPUM cohort, patients with tremor-dominant PD at the first visit had cNfL concentrations similar to those of the healthy controls, while patients with mixed or PIGD subtypes had higher concentrations (median difference 238 ng/L, *p* < 0.05 and 440 ng/L, *p* < 0.05, respectively). However, after adjustment for age and sex, patients with all PD phenotypes had significantly higher cNfL concentrations than the controls. The cNfL concentration also differed in relation to the major presenting symptom in the validation cohort (table 3), with patients presenting with balance impairment or gait difficulty (n = 30) having a higher cNfL concentration than patients presenting with bradykinesia (*p* < 0.05) or resting tremor (*p* < 0.01). These differences remained significant after age adjustment. In patients with repeated measurements, cNfL did not change significantly between baseline and the 1-year follow-up but increased between the 1- and 3-year follow-ups (median increase 98 ng/L, *p* = 0.030).

In the NYPUM cohort, the follow-up was complete until 8 years. Of the 99 patients with CSF samples in this cohort, 13 (13.1%) had died at 5 years and 27 (27.3%) at 8 years. Nine patients died during follow-up in the validation cohort. The most common cause of death in all patients with PD was pneumonia, consistent with a previous study.^4^ Other causes were described in the same study. A higher cNfL concentration at baseline predicted a shorter survival in the NYPUM cohort (*p* < 0.001), and this finding was confirmed in the validation cohort (*p* < 0.001). The hazard ratio (HR) for death during follow-up increased 1.36 times in the NYPUM cohort and 3.57 times in the validation cohort per 1–ng/mL increase in cNfL (table 4). Considering all 293 patients in the pooled PD cohort, cNfL concentrations above the overall median of 903 ng/L around the time of diagnosis (as measured by the baseline sample) conferred a 5.8 times higher hazard for death during follow-up (95% confidence interval 2.82–11.85, *p* < 0.001; table 4 and figure 1). Among patients with a low baseline cNfL concentration, below 965 ng/L and 925 ng/L (which were the cutoffs with the highest Youden index, and similar to the levels in the controls), 100% and 91% of the patients survived during the next 5 or 8 years, respectively. The AUROC for predicting survival by the cNfL concentration was slightly higher at the earlier (AUROC 0.79, *p* < 0.001) compared to the later (AUROC 0.75, *p* < 0.001) time point.

**Table 4:**
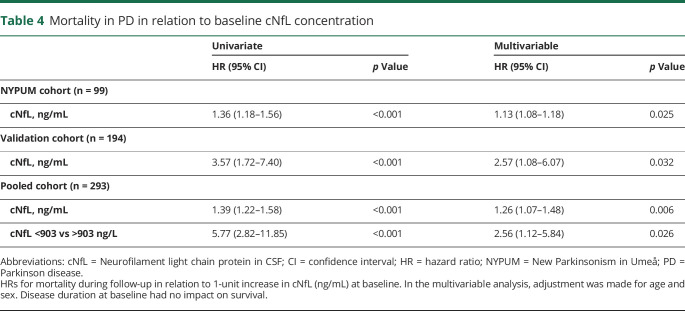
Mortality in PD in relation to baseline cNfL concentration

### Relative impacts of age and cNfL on disease severity

Older age in PD correlated with higher cNfL concentration (increasing ≈38 ng/L per year). However, all associations between cNfL and clinical features and survival remained significant after adjustment for age. An increase in 1 unit of cNfL at baseline (in ng/mL) in the pooled PD cohort was associated with an increase in the HY stage by 0.2 (*p* < 0.001), in the UPDRS Part III score by 4.1 points (*p* < 0.001), and in the total UPDRS score by 3.9 points (*p* < 0.001). The increases in clinical scores were somewhat lower after adjustment for age and sex (table 2). However, as shown by higher standardized β values in the multivariable models, the cNfL concentration was a stronger predictor of UPDRS scores than age. The standardized β for cNfL was 0.24 (*p* < 0.001) and for age was 0.13 (*p* = 0.012) in the prediction of the UPDRS Part III score and 0.21 (*p* = 0.002) and 0.18 (*p* = 0.010), respectively, in the prediction of the total UPDRS score (showing a stronger impact of cNfL than of age). Age contributed slightly more than cNfL to predict the HY stage, but cNfL also contributed significantly.

The association between high baseline cNfL and shorter survival in PD remained significant after adjustment for age and sex in both cohorts, as well as in the pooled cohort (table 4). In the validation cohort, high baseline cNfL was a stronger predictor for death during follow-up than was the age of the patient, rendering age at baseline a nonsignificant covariate (*p* = 0.183).

### cNfL in relation to brain imaging

**Figure 2:**
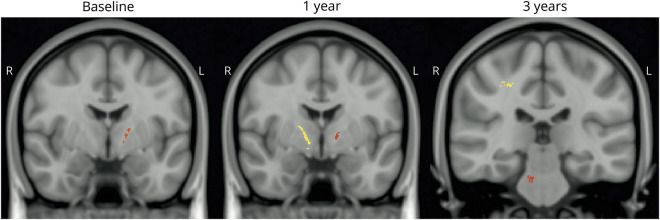
Imaging of impaired diffusion associated with cNfL elevations in PD Brain diffusion tensor imaging of patients in the New Parkinsonism in Umeå (NYPUM) cohort. Imaging was performed in 45, 20, and 14 patients with Parkinson disease (PD) at baseline (left) and the 1-year (middle) and 3-year (right) follow-ups, respectively. For all contiguous diffusion tensor imaging clusters, the fractional anisotropy value in all individual voxels significantly correlated with the neurofilament light chain protein in CSF (cNfL) level after adjustment for age and sex. A more yellow color denotes a larger cluster.

The severity of striatal ^123^I-FP-CIT uptake deficits was positively correlated with the cNfL concentration at baseline (table 5). The strongest correlation was found in the right hemisphere and in the caudate nucleus. The anatomic locations where axonal fiber disintegrity, as measured by the FA value derived from DTI, correlated with a higher cNfL in clusters with at least >30 voxels are shown in table 5. Associations between cNfL concentration and axonal lesions on DTI were found, e.g., in the anterior and posterior limb of internal capsule unilaterally (at the right side) at the level of the thalamus at baseline. This finding was more pronounced and found bilaterally at a similar location after 1 year, together with several cNfL-associated lesions on DTI in frontal lobe axonal tracts in proximity to the cortex, and at 3 years in the pons and, among other locations, the limbic lobe (table 5 and figure 2).

**Table 5:**
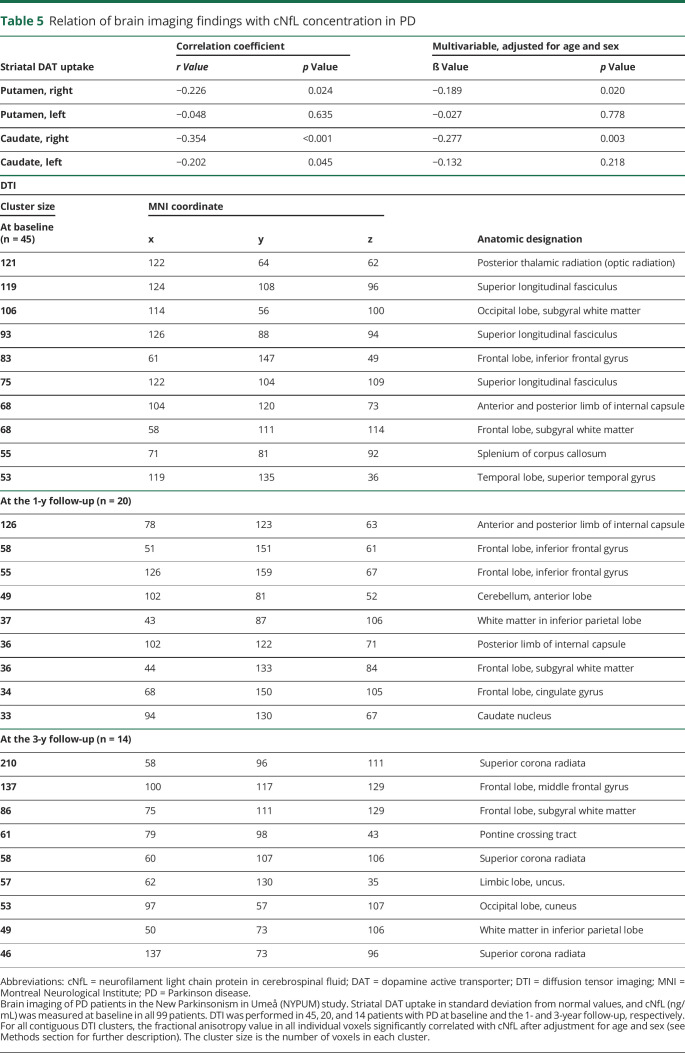
Relation of brain imaging findings with cNfL concentration in PD

## Discussion

In this population-based and clinical study of incident PD, we investigated whether high cNfL levels in the early disease phase reflect disease severity and risk of increased mortality at follow-up. We show that higher cNfL was related to more severe PD symptoms, as measured by clinical scales, and shorter survival in the population-based NYPUM cohort, as well as independently, in the validation cohort. These relationships were evident although the absolute cNfL values at the group level were not particularly high in PD compared with many other neurodegenerative diseases such as frontotemporal dementia or amyotrophic lateral sclerosis.^7^ At the subgroup level, however, the cNfL values were higher in patients with PD presenting with postural instability or gait difficulty compared with patients with other phenotypes of PD.

In the population-based NYPUM cohort, all patients with incident idiopathic parkinsonism in the studied area, rather than the referred cases, were included to avoid selection bias. This PD cohort should therefore provide information that is generalizable to the real-life experience of the population with idiopathic PD. Of note, some differences between the population-based and validation cohorts (consisting of the referred, consecutive patients with new-onset PD) were found. The HY stage and disease severity as measured by the UPDRS were higher in the population-based cohort. The cNfL level in the population-based cohort was higher than in the validation cohort, and this was not explained by the minor age difference. Even so, the relationships between higher cNfL, increased PD severity, and higher all-cause mortality were similar in both cohorts.

In the validation cohort, the follow-up period was shorter and there were fewer fatalities than in the NYPUM cohort, but the increase in the HR for death per unit of baseline cNfL increase (HR 3.6) was higher. cNfL was a stronger predictor of death during follow-up than age in this cohort. These characteristics may indicate that cNfL is a particularly sensitive marker of increased mortality in early PD.

The reason for the marked disease heterogeneity in PD is largely unknown but could relate to differences in the pattern or extent of neurodegeneration.^26–28^ Tremor-dominant PD is consistently associated with a more benign prognosis and less striatal dopamine depletion compared with non–-tremor-dominant phenotypes.^29–31^ The tremor-dominant phenotype is also associated with a lower risk of dementia, less cortical pathologies, and a longer life expectancy.^4,5,32^ In the present study, more severe bradykinetic-rigid or axial symptoms, postural instability, and gait impairment were related to higher cNfL, while tremor was unrelated. This may be consistent with less nigrostriatal degeneration and less cortical pathology in tremor-dominant PD. Our results add to the hypothesis that resting tremor may reflect a relatively benign circuit dysfunction in PD.^5^

DTI is an MRI technique that measures water diffusivity in white matter axon tracts. FA represents the directionality of diffusion, which is particularly sensitive to microstructural integrity in axons.^33^ Axonal tract degeneration is detectable early in PD, and there is evidence that DTI changes precede changes observed on conventional, structural MRI such as atrophy.^14^ Brain areas that have shown altered diffusion in PD include the substantia nigra and thalamic projections, the projection fibers from the substantia nigra to the striatum, and subcortical-cortical, callosal, and cortical pathways such as the superior longitudinal fasciculus.^33–39^

cNfL-correlated diffusion impairments in this study were found, among other locations, in internal capsule axon tracts unilaterally (at the right side) at the level of the thalamus at baseline and after 1 year more markedly and bilaterally in a similar location. At 3 years, such cNfL-associated lesions were found in the right-sided superior corona radiata (possibly in axon tracts extending from the internal capsule) in proximity to the frontal cortex and in the cingulum and pons. This longitudinally developing pattern of cNfL associated axon fiber disintegrity on DTI may reflect a progressive, spreading pathology in PD. Although the resolution limitations call for some caution, the observed pattern could suggest the spread of α-synuclein disease pathology in connected axonal projecting systems of the brain, with a predominant caudo-rostral course. The early finding (at baseline and the 1-year follow-up) of several cNfL-associated lesions in association fiber tracts of the cerebral hemispheres such as the superior longitudinal fasciculus and axon tracts of the frontal lobe is in agreement with previous findings in PD.^38,39^

SPECT and PET studies in PD consistently show more prominent denervation of dopaminergic neurons in the putamen than in the caudate nucleus. The caudate nucleus is affected later, as a result of disease progression.^21,40^ Early denervation in the caudate nucleus, which was related to higher cNfL in the present study, may therefore be a more sensitive marker of rapidly progressive neurodegeneration compared with the putamen. Impaired function of the caudate nucleus is also related to a higher risk of developing dementia and a shorter life expectancy in PD.^8,41^ The reason for the possible right-sided disease predominance in our imaging data is unknown, but this predominance also was observed in a previous study.^41^

Our center has extensive experience in using DTI for visualizing neuronal fiber disintegrity.^37,42^ Even so, it is not clear exactly what the cNfL-related white matter lesions on DTI represent. They may represent pathologic processes other than the α-synuclein proteopathy of PD. NfL concentration is also, for instance, increased by ischemic small vessel disease^43^ and Alzheimer disease.^44^ Comorbid brain pathology such as ischemic small vessel disease or Alzheimer disease pathology could contribute to increased mortality in PD and DTI findings and may relate to balance and gait impairment in PD.^45,46^ However, the finding that increased cNfL is associated with lower striatal ^123^I-FP-CIT uptake, higher scores in validated PD severity scales, and hyposmia indicates that the increase of cNfL is at least partially specific for PD neurodegeneration. Furthermore, the cNfL-related lesions on DTI were not found in areas typically affected by chronic, ischemic vascular injury such as in the periventricular white matter.^47^ Hence, the cNfL levels appear to reflect the intensity of the neurodegenerative process of PD. In parkinsonism, high cNfL could increase the suspicion of either a more aggressive form of PD or (if atypical features are present) atypical parkinsonism. Because of the close correlation between cNfL and NfL measured in blood, the biomarker qualities of cNfL are also likely to pertain to even more accessible blood NfL.^48,49^

The possibility of uncontrolled confounding factors and the limited number of neuropathologic diagnoses and healthy control participants are further limitations of this observational study. The risk of incorrect PD diagnosis was nonetheless minimized by long follow-up by specialized neurologists at a movement disorders unit and the finding of pathologic uptake on DAT imaging in all patients. The population-based and prospective study designs are strengths. The possibility of unspecific findings on DTI, as discussed above, limits inferences in relation specifically to PD pathology. However, the repeat DTI in patients with PD enables a search for disease patterns that are likely to change over time.

No validated neurochemical biomarker exists in PD to measure disease activity and to predict prognosis. NfL is likely to have clinical utility in PD for several purposes, e.g., as shown by the ability to predict disease severity and long-term survival in 2 different PD cohorts. NfL may enhance the selection of patients with PD in clinical trials by enrichment of patients with a faster disease progression. Further research is needed to determine whether NfL in CSF or blood can be used to longitudinally monitor the progression of PD or whether NfL increases can be reversed by neuromodulatory therapy.

## Glossary

AUROC: area under the receiver operating characteristic curve
cNFL: NfL in CSF
DAT: dopamine transporter
DTI: diffusion tensor imaging
FA: fractional anisotropy
HR: hazard ratio
HY: Hoehn and Yahr
NfL: neurofilament light chain protein
NYPUM: New Parkinsonism in Umeå
PD: Parkinson disease
PIGD: postural imbalance and gait difficulty
UPDRS: Unified Parkinson's Disease Rating Scale
